# Rotational atherectomy via retrograde popliteal access without embolic protection for complex femoropopliteal lesions

**DOI:** 10.1186/s42155-026-00745-0

**Published:** 2026-07-29

**Authors:** Onur Taydas, Erbil Arik, Mehmet Ali Durmus, Muhammed Said Besler, Ismail Ozer, Ahmet Yasin Kabas, Volkan Tasci, Omer Faruk Topaloglu, Fahrettin Yusuf Altintas, Mustafa Ozdemir, Mehmet Halil Ozturk, Bulent Arslan

**Affiliations:** 1 Faculty of Medicine, Department of Radiology, Sakarya University, Sakarya, Türkiye; 2 Faculty of Medicine, Department of Radiology, Marmara University, Istanbul, Türkiye; 3 Faculty of Medicine, Department of Radiology, Istanbul Medeniyet University, Istanbul, Türkiye; 4 Department of Interventional Radiology, Rush University Medical Center, Chicago, IL USA

**Keywords:** Retrograde popliteal access, Rotational atherectomy, Chronic total occlusion, Femoropopliteal disease, Drug-coated balloon, Distal embolization

## Abstract

**Background:**

Our study aims to evaluate the technical feasibility and safety of retrograde popliteal artery access (RPA) combined with rotational atherectomy and drug-coated balloon (DCB) angioplasty for complex femoropopliteal lesions, specifically without the use of distal embolic protection devices (EPD).

**Methods:**

This single-center, single-operator, retrospective study enrolled 22 consecutive patients with TASC B and C femoropopliteal lesions treated between January 2021 and June 2025. All had severe claudication (Rutherford class 3) and calcified lesions. All underwent ipsilateral retrograde popliteal access, JetStream rotational atherectomy, and Ranger DCB post-dilatation (Boston Scientific) without embolic protection. Technical success, access-site safety, ankle–brachial index (ABI), and 12-month patency were evaluated.

**Results:**

Mean age was 69.9 ± 7.9 years; 18 (81.8%) were male. Technical success was 95.5% (one bailout stent). No embolic events, procedure-related deaths, amputations, or major complications occurred. One patient (4.5%) had a minor access-site hematoma. At 12 months (19 patients), Kaplan–Meier analysis demonstrated a primary patency rate of 89.9% and a secondary patency rate of 100% (as all TLRs were successful). Two patients required clinically driven target lesion revascularization (TLR) at 6 and 10 months; freedom from TLR at 12 months was 89.9%. Mean ABI improved from 0.47 ± 0.07 to 0.85 ± 0.11 at 1 month (P < .001) and remained stable at 12 months (0.86 ± 0.04).

**Conclusions:**

RPA combined with rotational atherectomy and DCB angioplasty without embolic protection was technically feasible and did not result in clinically detectable macroembolization. However, definitive conclusions regarding microembolic safety cannot be drawn from these data alone.

**Graphical Abstract:**

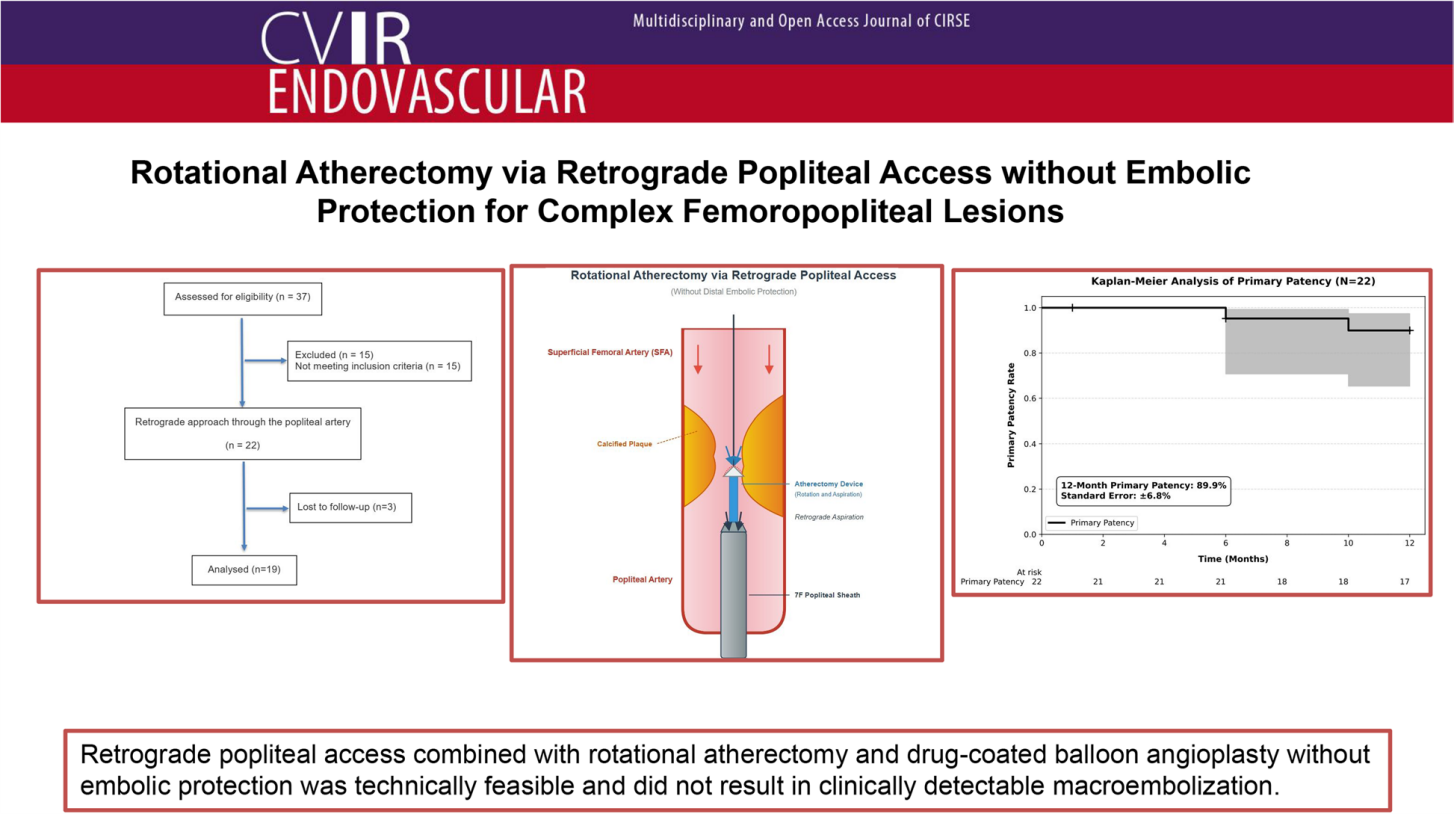

## Introduction

Peripheral arterial disease (PAD) is a clinical manifestation of systemic atherosclerosis. It affects more than 200 million people worldwide and is associated with significant morbidity and mortality [1]. The clinical presentation of the disease differs based on several factors, including the specific locations affected, the severity of atherosclerotic plaque buildup, the number of blood vessels involved, and how well collateral blood flow is maintained to the lower limbs. Symptoms can range from intermittent claudication to critical limb ischemia [2]. Revascularization is crucial for managing advanced PAD, as it reduces the risk of amputation and enhances quality of life [3].

Retrograde popliteal access is a valuable technique in complex femoropopliteal interventions. It is particularly useful after failed attempts to cross the lesion antegrade. This approach is beneficial in cases of a hostile common femoral artery, when iliac occlusive disease prevents access from the contralateral side, and in heavily calcified chronic total occlusions (CTOs). Utilizing retrograde access can enhance the chances of successfully navigating through the true lumen and delivering devices effectively [4]. When antegrade procedures fail, retrograde access through the popliteal artery (RPA) has become an essential technique for successfully crossing these lesions [5].

Traditional revascularization methods often relied on plain balloon angioplasty and bare-metal stenting. However, these techniques frequently face limitations due to high rates of neointimal hyperplasia and in-stent restenosis, which can exceed 40% in long-segment lesions [6]. To enhance vessel patency, a "debulk and deliver" strategy has gained popularity. This approach combines rotational atherectomy for plaque modification with drug-coated balloon (DCB) angioplasty. Although rotational atherectomy is effective in treating calcified segments, the risk of distal embolization often requires the use of embolic protection devices (EPDs). However, using EPDs in a retrograde transpopliteal workflow adds significant technical complexity and may not be anatomically feasible in all cases [7].

This study aims to evaluate the feasibility, procedural safety, and 12-month outcomes of a stent-sparing, EPD-free atherectomy and DCB strategy via the RPA for complex femoropopliteal disease.

## Methods

### Study design and patient selection

Ethical approval for this retrospective study was obtained from the local ethics committee (E-43012747–050.04–481649-365/June 17, 2025). Owing to its retrospective design, the requirement for written informed consent was waived.

**Fig. 1:**
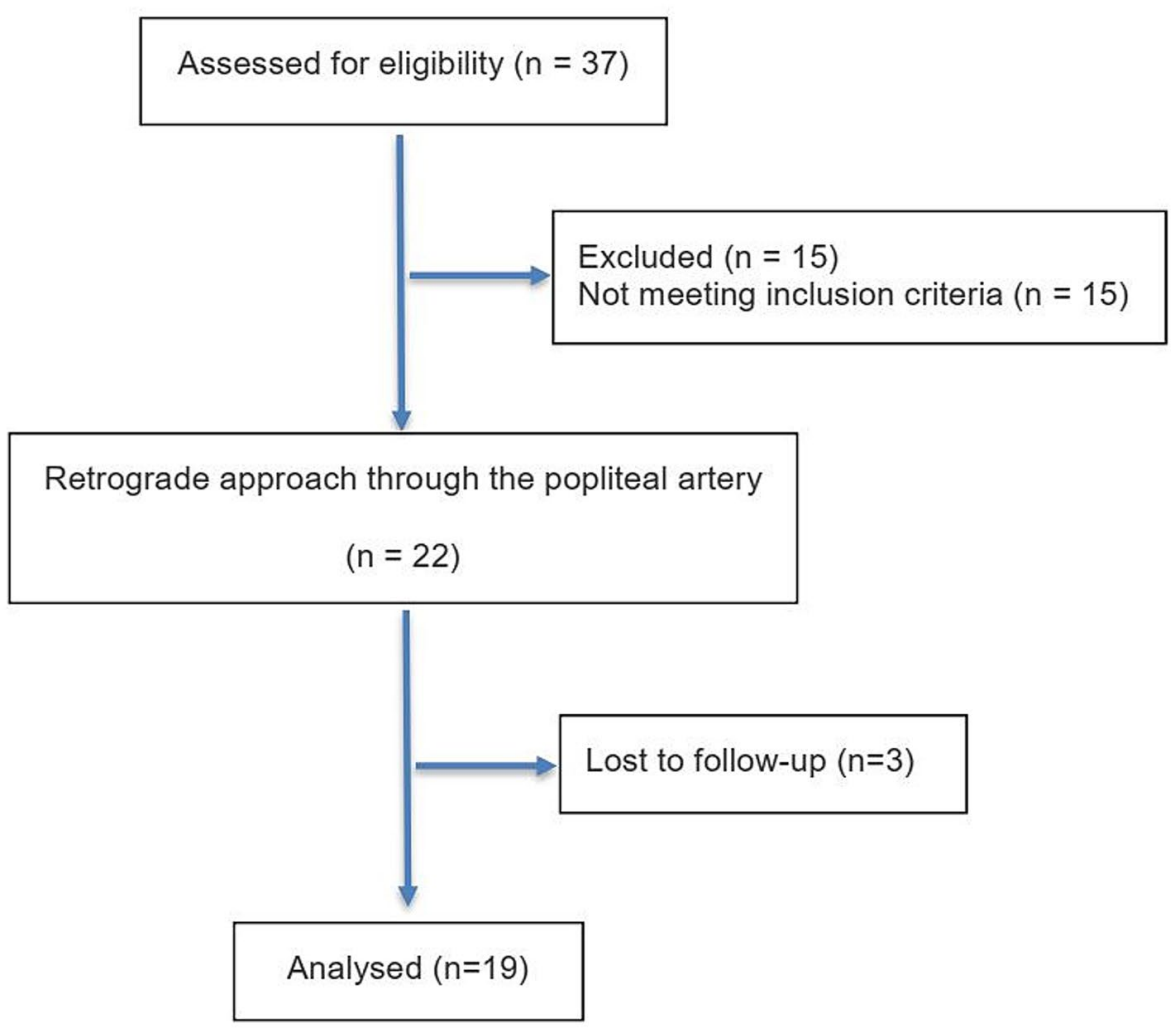
Flowchart of the study

This study was conducted as a single-center, retrospective analysis involving 22 consecutive patients (mean age 69.9 ± 7.9 years, with 18 men). These patients were treated for TASC B and C lesions located in the common and superficial femoral arteries, as well as in the above-knee popliteal arteries (P1 segment), at our interventional radiology unit between January 2021–June 2025 (Fig. 1). Calcification severity was graded using the Peripheral Arterial Calcium Scoring System (PACSS).

Patients over 18 years with intermittent claudication (Rutherford classification 3) and calcified lesions in the superficial femoral and P1 popliteal segments were included in this study.

Patients were excluded from the study if they met any of the following criteria: (1) significant aortoiliac occlusive disease that required treatment prior to undergoing femoropopliteal intervention; (2) acute limb ischemia (Rutherford category 6); (3) non-atherosclerotic arterial diseases, such as vasculitis or thromboangiitis obliterans; or (4) contraindications to atherectomy or drug-coated balloon angioplasty.

Patients were excluded for the following reasons (*n* = 15): significant uncorrected aortoiliac occlusive disease (*n* = 8), acute limb ischemia/Rutherford 6 (*n* = 5), and non-atherosclerotic arteritis (*n* = 2).

### Lesion assessment and procedural technique

Before the procedure, all patients underwent preprocedural computed tomography angiography (CTA) baseline Doppler ultrasound for evaluation. Based on preprocedural imaging findings, the length and maximum degree of stenosis were analyzed. Baseline demographic and clinical characteristics were recorded. Lesion characteristics, including lesion length, anatomical location, and the presence of occlusion, were assessed angiographically. Lesions were classified based on the TransAtlantic InterSociety Consensus (TASC II) criteria. Calcification burden was formally graded using the Peripheral Arterial Calcium Scoring System (PACSS).

All procedures were carried out by an operator who has more than 10 years of experience. All patients underwent ipsilateral retrograde popliteal artery access in the prone position. This method was chosen because antegrade access through the ipsilateral common femoral artery was not possible due to either proximal SFA occlusion or critical stenosis at the planned access site. Additionally, contralateral femoral access was not an option because of aortoiliac occlusion or severe stenosis. Other factors considered included the facilitation of lesion crossing in cases of complex SFA disease and the potential reduction of embolic burden through retrograde flow dynamics and sheath aspiration.

Under ultrasound guidance, a 7-F sheath (ShunGuider, Shunmei, Shenzen, China) was inserted into the popliteal artery. Immediately after placing the sheath, intravenous unfractionated heparin (Koparin, Kocak, Türkiye) was administered to maintain an activated clotting time of over 250 s. This treatment was necessary before proceeding with lesion crossing and atherectomy. Initial angiography was performed to evaluate the below-the-knee run-off, followed by imaging of the femoropopliteal segment.

Intraluminal crossing of the lesion was achieved using a 0.035-inch hydrophilic guidewire (Zipwire, Boston Scientific, Marlborough, MA, USA) in conjunction with a 0.035-inch support catheter (Rubicon, Boston Scientific, Marlborough, MA, USA). This setup was then replaced with a stiff 0.014-inch guidewire (Thruway, Boston Scientific, Marlborough, MA, USA). Completion angiography confirmed successful intraluminal crossing, and multiple oblique views were utilized to verify the central positioning and straight shape of the guidewire, as opposed to a spiral shape which would indicate subintimal crossing.

**Fig. 2:**
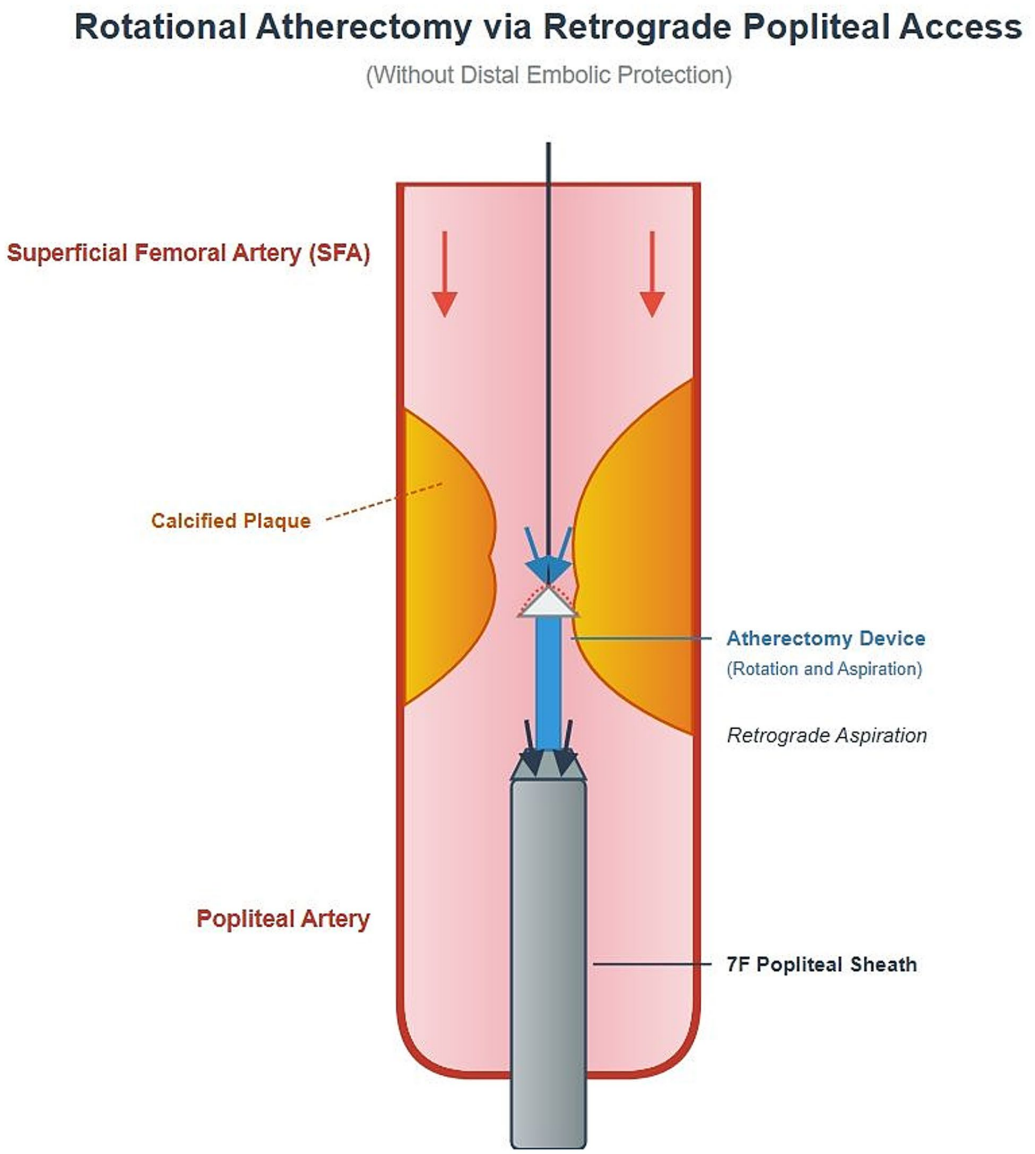
The illustration provides a summary of the intervention

Rotational atherectomy was performed using the Jetstream™ Atherectomy System (2.4/3.4 mm cutter configuration) (Boston Scientific, Marlborough, MA, USA), a device that combines rotational cutting and aspiration capabilities. The device was advanced slowly at a rate of approximately 1 mm/s, following the manufacturer’s instructions for use. Controlled plaque debulking was carried out against the direction of blood flow under fluoroscopic guidance. Typically, two passages with the blades down and two with the blades up were performed. Distal embolic protection devices were not utilized (Fig. 2). After completing atherectomy, sheath aspiration was routinely performed to minimize embolic burden. A 50-mL syringe was connected to the side port of the 7 F popliteal sheath, and 20–30 mL of blood was rapidly aspirated and macroscopically inspected for atherosclerotic debris prior to standard flushing. All patients underwent post-dilatation after plaque debulking at the target lesion level using an appropriately sized drug-coated balloon (Ranger, Boston Scientific, MA, USA). Completion angiography was routinely performed to evaluate residual stenosis, identify potential dissections, and assess distal tibial run-off.

After the removal of the sheath, hemostasis was achieved through manual compression lasting 10 to 15 min. Following the procedure, patients were prescribed dual antiplatelet therapy (DAPT) consisting of acetylsalicylic acid (ASA) and clopidogrel. In addition, our institutional protocol required a 10-day course of prophylactic low-molecular-weight heparin (LMWH). Although current guidelines recommend DAPT alone [2], this LMWH protocol aims to reduce the perceived risk of early access-site thrombosis following the placement of a 7 F sheath in affected popliteal arteries. Furthermore, mechanical atheroablation via the Jetstream device induces extensive endothelial denudation, leaving a highly pro-thrombotic raw surface in the immediate post-procedural phase. To protect the alternative access site from acute mechanical occlusion and maintain patient safety during early vessel healing, this temporary LMWH bridging was deemed clinically necessary. Patients will then continue with lifelong ASA monotherapy.

### Follow-up and definitions

Patients were regularly scheduled for clinical and Doppler ultrasound follow-ups at 1, 6, and 12 months after the procedure. When available, additional follow-ups were conducted beyond the 12-month mark. The median follow-up period was 14 months (range 8–22). Ankle–brachial index (ABI) measurements were taken at each follow-up visit. Further evaluations were conducted if any symptoms arose during the procedure. Angiography was performed if there was suspicion of occlusion or critical stenosis.

Technical success was defined as the successful completion of the intended endovascular procedure, resulting in restored antegrade flow and residual stenosis of less than 30%, without any flow-limiting dissection.

Clinical success was defined as an improvement in symptoms and/or Rutherford category, without the need for immediate target lesion revascularization (TLR).

Distal embolization was characterized by the emergence of a new angiographic intraluminal filling defect associated with flow disturbance, which required adjunctive aspiration or catheter-based intervention during or after atherectomy.

Primary patency was defined as the absence of clinically driven TLR during the follow-up period. Secondary patency was established as the restoration of vessel patency following a repeat endovascular intervention after the loss of primary patency.

Procedure-related complications were classified according to the modified CIRSE grading system [8].

### Statistical analysis

Due to the exploratory and observational nature of this study, a formal sample size calculation was not conducted. Statistical analyses were performed using MedCalc (version 12, Ostend, Belgium). Descriptive statistics are presented as median (minimum–maximum) and mean ± standard deviation. Categorical variables are expressed as frequencies and percentages. The independent-samples t-test was employed to compare continuous variables that followed a normal distribution, while the Mann–Whitney U test was used for data that did not conform to normal distribution, as determined by the Shapiro–Wilk test. Changes in ankle-brachial index (ABI) across the three time points (baseline, 1 month, 12 months) were evaluated using repeated-measures analysis of variance (ANOVA) performed strictly on the 19 patients with complete longitudinal follow-up data.Sphericity was assessed using Mauchly’s test, and a Greenhouse–Geisser correction was applied where the assumption was violated. Primary and secondary patency, as well as freedom from clinically driven target lesion revascularization (TLR), were estimated using the Kaplan–Meier method. It is noted that primary patency loss and TLR were co-primary endpoints defined identically in this series; the equivalent rates (89.9%) reflect that both TLR events resulted in patency loss, confirming these were not coincidentally identical values. All tests were two-sided, with statistical significance defined as P < 0.05. 95% confidence intervals are reported throughout; readers are cautioned that the wide intervals observed reflect the limited statistical power of this small feasibility series and should not be interpreted as definitive estimates of treatment efficacy.

## Results

### Patient lesion characteristics

All patients experienced severe claudication (Rutherford class 3). The analyzed cohort comprised heavily calcified lesions (calcification severity was ordinally categorized according to the PACSS grading system: 13 patients (59.1%) had PACSS Grade 3, and 9 patients (40.9%) had PACSS Grade 4). Among the patients, 10 (45.5%) had a TASC B lesion, while 12 (54.5%) had a TASC C lesion. The mean lesion length was 16.6 ± 8.0 cm (median, 16 cm; range, 2.5–32 cm). Patient characteristics are detailed in Table 1. Comprehensive baseline angiographic data, including superficial femoral artery (SFA) reference vessel diameter (RVD), popliteal artery diameters, and infrapopliteal run-off vessel status, are presented in Table 2.

**Table 1:** Patient characteristics and lesion data

Variable	Value (*n* = 22)
Age (years), Mean ± SD	69.9 ± 7.9
Sex (M/F)	18/4
Diabetes Mellitus, n (%)	11 (50.0%)
Hypertension, n (%)	9 (40.9%)
Active Tobacco Use, n (%)	12 (54.5%)
Coronary Artery Disease, n (%)	14 (63.6%)
Baseline ABI, Mean ± SD	0.47 ± 0.07
Lesion Length (cm), Mean ± SD	16.6 ± 8.0
PACSS Grade 3, n (%)	13 (59.1%)
PACSS Grade 4, n (%)	9 (40.9%)

**Table 2:** Baseline angiographic data

Variable	Value
SFA Reference Vessel Diameter (mm), Mean ± SD	5.4 ± 0.6 mm
Popliteal Artery Reference Diameter (mm), Mean ± SD	5.8 ± 0.5 mm
Patent Run-off Vessels (0/1/2–3), n	0/5/17
Chronic Total Occlusion (CTO), n (%)	14 (63.6%)

### Procedural outcomes and safety

Mean procedure duration was 61.1 ± 27.1 min, mean fluoroscopy time was 17.7 ± 11.1 min, and mean contrast load was 70 ± 30 ml.

Technical success was achieved in 95.5% (21/22) of cases. One patient (4.5%) experienced a severe flow-limiting dissection after DCB inflation, necessitating the deployment of a bailout self-expanding nitinol stent (6 × 80 mm, Epic, Boston Scientific, MA, USA) that achieved a satisfactory final angiographic result. Upon macroscopic inspection of the aspirated blood via the 7 F side port, visible macroscopic debris was observed in 0 cases. Consequently, no clinically evident embolic events, such as abrupt loss of run-off or acute limb ischemia, were identified during completion angiography. However, subclinical microembolization cannot be ruled out, as dedicated post-procedural multi-projection tibial run-off imaging and filter-capture analyses were not conducted.

Access-site safety was excellent despite the use of the 7-F system. Routine Doppler ultrasounds conducted every month confirmed that there were no instances of pseudoaneurysms or fistulas in any of the patients. One patient (4.5%) experienced a minor access-site hematoma (CIRSE Grade 2), but it resolved with conservative management. No major complications (CIRSE Grade ≥ 3) were observed. There were no mortalities or major amputations within 30 days.

### Clinical outcomes and patency

**Fig. 3:**
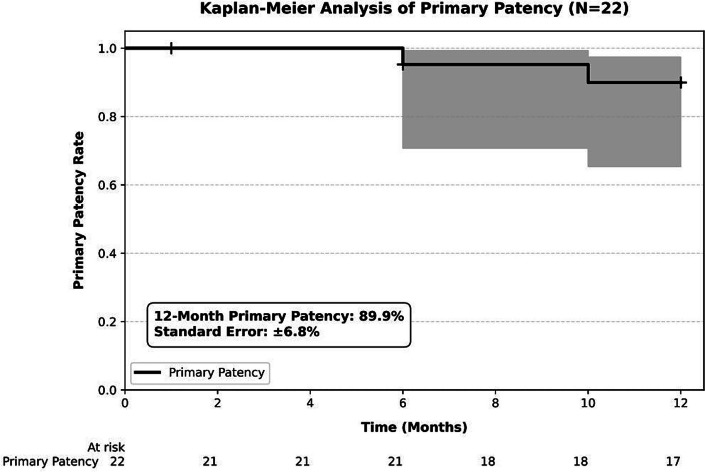
Kaplan–Meier analysis of primary patency rates

Three patients (13.6%) were lost to follow-up before the 12-month mark due to relocation (censored at 1, 6, and 6 months), and these patients were censored in the survival analysis. In the remaining 19 patients analyzed, the mean ankle-brachial index (ABI) showed a significant improvement, reaching 0.85 ± 0.11 at 1 month (*P* < 0.001), and this improvement was maintained at 12 months with a value of 0.86 ± 0.04. The Kaplan–Meier analysis indicated a primary patency rate of 95.2% (SE: ± 4.6%) at 6 months and 89.9% (SE: ± 6.8%) at 12 months. Two patients experienced symptom recurrence and required target-lesion revascularization (TLR) at 6 and 10 months. Both TLRs were successful, resulting in a secondary patency rate of 100%. Furthermore, the freedom from clinically driven TLR at 12 months was identical to primary patency at 89.9% (Fig. 3).

## Discussion

The main finding of this retrospective feasibility study is that treating complex, heavily calcified femoropopliteal lesions using rotational atherectomy and drug-coated balloons via retrograde pedal access—without the routine use of embolic protection devices—is technically feasible. This approach did not lead to any clinically detected macrovascular complications in a selected group of patients. Our findings align with a recent study conducted by Valerio et al. Their multicenter research reported a 12-month freedom from target lesion revascularization (TLR) following the treatment of TASC B and C femoropopliteal lesions using a rotational atherectomy device and drug-coated balloons (DCB). A key distinction between the two studies is the access site: while they routinely utilized femoral access, we consistently employed retrograde popliteal access [9].

The decision to exclude EPDs from our cohort is based on two main factors. First, using EPDs in a retrograde transpopliteal workflow presents significant spatial constraints and adds technical complexity. Second, we hypothesized that the retrograde hemodynamic flow configuration, combined with the aspiration function of the rotational atherectomy device and our protocol of vigorous manual sheath aspiration (using a 50-mL syringe directly from the 7 F side port), may effectively remove a substantial amount of the debris generated. Furthermore, the insertion of a large-bore 7 F sheath into a diseased popliteal artery creates relative flow stagnation (a partial “flow-stop” effect). We hypothesize that this relative stasis plays a crucial role in preventing debris from being carried downstream, thereby facilitating high-yield aspiration. It is important to highlight that the lack of acute limb ischemia does not imply a true embolization rate of 0%. Valerio et al. reported a distal embolization rate of 5.7% with this combination of devices [9]. In our study, we did not use distal filters to capture microdebris, nor did we conduct thorough, multi-level post-procedural imaging to assess run-off. As a result, subclinical microembolization may have been underestimated [10].

Distal embolization during peripheral atherectomy has shown significant variability across various contemporary studies. The rates of embolization differ based on the type of device used and the complexity of the lesions, with higher rates frequently observed in complex, heavily calcified lesions [10]. These findings underscore the risk of embolic events associated with plaque removal procedures. As a result, some authors recommend the routine use of distal embolic protection devices during atherectomy for treating femoropopliteal lesions [11]. A large-scale study revealed that using distal embolic protection devices during atherectomy does not significantly impact patient outcomes. Additionally, these devices add extra cost and require more time [12]. Therefore, our technique suggests that performing atherectomy without distal embolic protection devices might be more practical.

Our strategy utilized ipsilateral retrograde popliteal access, allowing for atherectomy to be performed in a retrograde hemodynamic configuration. This setup, combined with the inherent aspiration capability of the Jetstream device and routine sheath aspiration after atherectomy, may effectively control embolic burden [13].

Ensuring safety at the access site is crucial when using a 7**-**F sheath in the popliteal artery [14]. Our protocol demonstrated safety, with only one minor hematoma reported, which was confirmed by a routine Doppler ultrasound conducted 1 month later. Additionally, our institution has adopted a post-procedural regimen of LMWH for 10 days, which is a deliberate deviation from the most recent ESVS guidelines that recommend DAPT alone [2]. This choice was made to prevent early thrombosis at the access site and around the sheath; however, it introduces a known variable that may confound the outcomes related to vessel patency.

Atherectomy effectively reduces the atherosclerotic burden, especially in advanced lesions with heavy calcification. In cases of femoropopliteal disease, it serves as an adjunctive therapy for treating heavily calcified plaques and exophytic lesions. Severely calcified blood vessels present a significant challenge for endovascular therapy and are not adequately addressed with PTA alone. Preparing the vessel with atherectomy prior to balloon angioplasty or stenting may enhance outcomes in this patient group [15].

Additionally, a recent meta-analysis comparing the outcomes of atherectomy and stent placement for symptomatic atherosclerosis of the common femoral artery (CFA) showed satisfactory results for both methods, with no significant advantage of one technique over the other [16].

Our treatment strategy follows the “leave nothing behind” approach, which is increasingly favored in the endovascular treatment of peripheral arterial lesions [17,18].

In our study, all patients received a drug-coated balloon (DCB) treatment in combination with rotational atherectomy. The 12-month exploratory results from the prospective randomized JET–RANGER study showed high rates of freedom from target lesion revascularization (TLR) at the 1-year follow-up, along with a significant reduction in the need for bailout stenting following rotational atherectomy [19]. Additionally, the recent CORVUS study compared procedural complications, patency, and adverse events between a stent strategy and DCB treatment after using the rotational atherectomy device for severely calcified femoropopliteal lesions. The findings indicated that DCB treatment after rotational atherectomy demonstrated comparable safety—aside from an increased risk of distal embolization—and high efficacy in patients with severely calcified lesions [20]. This indicates that DCB may be a viable alternative to stents for revascularization. Additionally, our strategy significantly reduced the risk of distal embolization.

Even with a strategy that prioritizes vessel preparation, provisional stenting may still be necessary to address flow-limiting dissections or elastic recoil, as noted in one patient from our study. This observation aligns with previous research indicating that additional scaffolding is often required in a minority of complex femoropopliteal interventions, especially in cases involving long and heavily calcified lesions [21]. Proper lesion preparation is crucial in severely calcified disease, where atherectomy can enhance luminal expansion and improve the performance of drug-coated balloons.

Moreover, the success of the procedure and the safety of the access site are significantly influenced by the experience of the operator and the consistent use of ultrasound guidance [14]. These factors have been shown to increase efficiency and reduce complications when using retrograde popliteal access. While retrograde popliteal access offers a stable and direct pathway for crossing lesions, alternative retrograde methods, such as pedal or tibial access, may be beneficial in specific situations, particularly when distal re-entry or limb-salvage strategies are necessary [22].

## Limitations

This study has several limitations. First, its retrospective, single-center design and relatively small sample size may introduce potential selection bias and limit the statistical power of the results. Second, all procedures were performed by a single experienced operator, which could affect reproducibility and limit generalizability to other centers with varying levels of expertise. Third, the absence of a comparator group, such as an antegrade approach or distal embolic protection devices, prevents a direct assessment of relative efficacy and the potential for embolic risk reduction. Additionally, distal embolization may have been underdetected because routine post-procedural imaging beyond completion angiography was not conducted, and embolic protection filters were not utilized to capture microdebris. Furthermore, patency assessment relied on clinical follow-up and duplex ultrasound without independent core laboratory adjudication, which could introduce measurement variability. Lastly, the follow-up period was relatively short, leaving longer-term outcomes beyond 1 year uncertain. Furthermore, the routine administration of a 10-day post-procedural LMWH course introduces a significant systemic confounding variable. While this aggressive regimen was mandatory for access-site protection in our institutional protocol, it may have favorably influenced early vessel patency and 1-month ABI outcomes. This limits the direct generalizability of our results to centers strictly adhering to guidelines recommending antiplatelet monotherapy or DAPT alone. To validate these findings and better define the role of retrograde atherectomy strategies without embolic protection in complex femoropopliteal disease, larger prospective multicenter studies with standardized imaging assessments and comparative cohorts are needed. Finally, out of 37 initially assessed patients, 15 were excluded (primarily due to severe aortoiliac disease or acute limb ischemia), creating a highly selected cohort which may introduce selection bias. Additionally, the lack of intravascular imaging (such as IVUS) limits our ability to comprehensively assess deep calcium morphological features and sub-angiographic dissections, which would have provided a more robust evaluation of lesion preparation.

## Conclusion

Our study demonstrated that a stent-sparing strategy utilizing retrograde popliteal access, rotational atherectomy, and drug-coated balloon angioplasty without embolic protection was technically feasible. This approach did not lead to clinically evident macroembolization or significant complications at the access site. By utilizing retrograde flow dynamics along with post-atherectomy sheath aspiration, it is possible to effectively manage embolic events without the routine application of distal embolic protection devices. This strategy, which avoids the use of stents, followed by drug-coated balloon angioplasty, may be a viable option for selected cases of anatomically challenging femoropopliteal disease, especially when antegrade access is not feasible. Larger, prospective multicenter studies incorporating standardized post-procedural tibial run-off imaging and comparative cohorts are required before this strategy can be broadly recommended.

## Data Availability

The datasets used and/or analyzed during the current study are available from the corresponding author upon reasonable request.

## Abbreviations

RPA: Retrograde popliteal artery access
DCB: Drug-coated balloon
EPD: Embolic protection devices
TASC: Trans-Atlantic Inter Society Consensus
ABI: Ankle brachial index
PAD: Peripheral arterial disease
CFA: Common femoral artery
SFA: Superficial femoral artery
PACSS: Peripheral Arterial Calcium Scoring System
DAPT: Dual antiplatelet therapy
TLR: Target lesion revascularization
CTO: Chronic Total Occlusion
